# Effects of IL‐18 on the proliferation and steroidogenesis of bovine theca cells: Possible roles in the pathogenesis of polycystic ovary syndrome

**DOI:** 10.1111/jcmm.16179

**Published:** 2020-12-07

**Authors:** Hong yuan Zhang, Fu fan Zhu, Ying jun Zhu, Yuan jing Hu, Xu Chen

**Affiliations:** ^1^ Tianjin Key Laboratory of Human Development and Reproductive Regulation Department of Gynecology Tianjin Central Gynecology and Obstetrics Hospital Affiliated to Nankai University Tianjin China; ^2^ Department of Obstetrics and Gynecology Second Xiangya Hospital of Central South University Hunan China

**Keywords:** bovine, IL‐18, polycystic ovary syndrome, proliferation, steroidogenesis, theca cells

## Abstract

Interleukin 18 (IL‐18) is a pleiotropic pro‐inflammatory cytokine and is associated with arrested follicle development and anovulation which are the typical pathological changes of PCOS. Theca cells (TCs) have a key role in follicular growth and atresia. But whether IL‐18 can directly affect ovarian TCs function is unknown. Therefore, the objective of this study was to determine the effect of IL‐18 on proliferation and steroidogenesis of bovine TCs and to explore the biological effect of IL‐18 on folliculogenesis. This work revealed that at 300‐1000 pg/mL, IL‐18 led to a time‐ and dose‐dependently increase in cell proliferation (*P* < .05). IL‐18 increased 17‐hydroxyprogesterone (17OHP4) and androstenedione (A2) secretion with up‐regulation of key steroidogenesis‐related genes CYP11A1 and CYP17A1 (*P* < .05). Furthermore, our data demonstrated that the IL‐18R protein is predominantly expressed in small‐follicle (3‐6 mm) TCs than large follicles (8‐22 mm) by immunohistochemistry. We also found that the stimulation effects of IL‐18 on TCs can be reversed with the addition of IL‐18BP as early as at 4 hours of culture and reached the peak at 16 hours. We conclude that IL‐18 appears to target TCs in bovine, and suggest an important role for this cytokine in ovarian function. Present findings further validate potential effects of IL‐18 in the conditions associated with follicular dysplasia and excessive growth of ovarian TCs (such as PCOS). But additional research is needed to further understand the mechanism of action of IL‐18 in theca cells as well as its precise role in folliculogenesis.

## 1 INTRODUCTION

Polycystic ovary syndrome (PCOS) is a complex disorder of the nervous‐endocrine‐metabolic network. At present, its pathogenesis is unclear. In recent years, evidence has accrued to support the concept that chronic “low‐grade” inflammation may contribute to the pathogenesis of PCOS. Many studies have reported that the expression of inflammatory factors, such as C‐reactive protein (CRP), interleukin IL‐6, and tumour necrosis factor‐a (TNF‐a), is higher in PCOS patients than in the normal population.^1^, ^2^, ^3^ Furthermore, the occurrence and development of PCOS have a close relationship with inflammatory factors.^4^, ^5^, ^6^

Interleukin 18 (IL‐18), formerly known as interferon‐γ inducing factor,^7^ is a pleiotropic, pro‐inflammatory cytokine, which plays a key role in the host defence against several infectious agents. IL‐18 modulates many genes involved in inflammation, infection and malignancy. Previous studies have shown that the follicular fluid (FF) of PCOS patients contains IL‐18 or its mRNA, and our recent study also showed that IL‐18 levels in FF of PCOS patients are higher than those in control women.^8^ It is likely that locally produced IL‐18 plays a physiological role in the pathogenesis of PCOS.

Typical pathological changes of PCOS include arrested follicle development and anovulation. IL‐18 has been suggested to affect ovarian folliculogenesis.^9^ Gutman et al^10^ described a high correlation between both pre‐ovulatory follicular fluid (FF) and serum levels of IL‐18 with the number of retrieved oocytes. Significantly higher IL‐18 levels were also detected in serum and peritoneal fluid of patients with severe ovarian hyperstimulation syndrome.^11^ Inactivation of IL‐18R with murine monoclonal antibody during ovarian stimulation reduced the number of ovulated oocytes and inhibited the expansion of cumulus cells surrounding the ovum.^12^

Theca cells (TCs), which surround the basal lamina, have a key role during follicular growth and atresia and synthesize androgens. Theca cells produce androgens from cholesterol through a series of reactions catalysed by steroid cytochrome P450 (CYP) hydroxylases and hydroxysteroid dehydrogenases under luteinizing hormone (LH) stimulation. Cholesterol is transported from the outer to inner mitochondria membrane by steroidogenic acute regulatory protein (StAR). Subsequently, steroidogenesis begins with conversion of cholesterol into pregnenolone by the cytochrome P450 side‐chain cleavage enzyme (CYP11A1). Pregnenolone is subsequently transferred to the cytoplasm where it is converted to androgens (androstenedione and dehydroepiandrosterone) by the catalytic actions of 3β‐hydroxysteroid dehydrogenase (3β‐HSD) and CYP17A1.

Abnormal function of T‐I cells has been shown to be associated with pathological conditions, such as PCOS. Hyperplasia of the ovarian theca cells compartment is a key feature of the polycystic ovary phenotype. Ovarian theca cells are recognized as one of the primary sources of excess androgen biosynthesis in women with PCOS,^13^ expressing a variety of genes encoding components of the steroidogenic pathway that are necessary for androgen and progestin biosynthesis.^14^ Ovarian hyperandrogenism is associated with the increased number of androgen‐producing cells contributing to increased androgen production,^15^ and then leading to anovulation.^16^

The biological functions of IL‐18 are mediated through its receptor. IL‐18 receptor (IL‐18R) is a member of the IL‐1 receptor family. It consists of a ligand‐binding domain (α‐chain), which binds IL‐18 with low affinity, and a signal‐transducing domain (β‐chain).^17^ Together, a formed high‐affinity IL‐18R complex transduces its signal to stimulate the MAPK pathway.^18^ Cytokines are increasingly recognized as potentially important local regulators of ovarian function.^19^ The expression of IL‐18R has been determined in theca cells, but the effect of IL‐18 on the proliferation and hormone secretion of TCs is not clear, and there is no related research report.

The aims of the present study were to: (a) examine the expression of IL‐18 signalling receptors in TCs across different stages of bovine antral follicle development; (b) use non‐luteinized bovine TC culture models to investigate whether IL‐18 affects steroid production; (c) explore the potential biological effects of IL‐18 on TCs proliferation in follicles sampled; (d) detect the effect of IL‐18 on the key enzymes in steroid hormone synthesis of TCs; (e) determine whether the effect of IL‐18 can be attenuated by IL‐18BP; and (f) explore the possible roles of IL‐18 in the pathogenesis of PCOS.

## 2 MATERIALS AND METHODS

### 2.1 Ethics statement

All procedures were reviewed and approved by the ethics committee of Tianjin Central Hospital of Gynecology Obstetrics, China (TJCHGO‐2018‐112). The local slaughterhouse in Tianjin provided ovaries for cattle aged 1‐2 years. The ovaries were dissociated according to the laboratory standards of Nankai University.

### 2.2 Isolation and culture of TCs

Bovine TCs were isolated from the ovaries of randomly cycling cattle obtained from the slaughterhouse. Freshly collected ovaries were placed on ice in saline with antibiotics (0.9% saline solution with 1% penicillin‐streptomycin) and transferred to the laboratory within 2 hours after collection. The follicles were dissected from the ovaries using surgical tweezers and striped until the surface was smooth. Follicles were then cut into halves under a stereomicroscope (Nikon) and the granulosa cells (GCs) were dislodged using a plastic inoculation loop. The follicle halves were shaken vigorously in a DMEM/F12 medium (GIBCO) to remove any remaining GCs. The theca internal layer was peeled away from the basement membrane and then was torn into small pieces, incubated for 30 minutes at 37°C with DMEM/F12 medium containing 1.0 mg/mL collagenase (170.0 U/mg, type 4; GIBCO), 0.2% glucose pH7.4 (Sigma‐Aldrich Inc), and 0.4% bovine serum albumin (GIBCO).^20^ The nondigested tissue was filtered out through sterile syringe filter holders with metal screens of 149 μm mesh. The isolated cells were pelleted twice in phosphate‐buffered saline (PBS) solution by centrifugation (800*g* for 10 minutes) to lyse any red blood cells.^21^ Cell viability was assessed by trypan blue exclusion and averaged above 90%. Then the TCs were re‐suspended in serum‐free medium containing collagenase (1.25 mg/mL) and DNase (0.5 mg/mL) to prevent cell clumping.^22^, ^23^ The resultant TCs preparations obtained using this method were judged to have <5% contamination with GCs.^24^

On average, 2.0 × 10^5^ viable cells were plated on 24‐well Falcon multiwell plates in 1 mL of medium and cultured in an environment of 38.5°C with 5% CO2 and 95% air in 10% FCS for the first 48 hours until cells reached 80% confluency. Cells were washed twice with 0.5 mL of serum‐free medium, treatments were applied in serum‐free medium for an additional 24 hours or 48 hours, and medium was either aspirated or collected from each well depending on the particular experiment. The concentration of LH was selected based on previous studies.^25^ This culture system was developed to yield hormonally responsive nonluteinized TC.^26^, ^27^ First, progesterone production does not increase with time using this culture paradigm.^27^ Secondly, the morphology of the TC retains a fibroblastic appearance.^28^ Third, the TC remain responsive to LH in terms of CYP17A1 mRNA and androstenedione production.^29^

### 2.3 Immunofluorohistochemistry detection of IL‑18R in TCs

The expression of IL‐18R on the TC was detected by immunofluorescence histochemistry (IF). The cells on the slide were fixed in 10% formalin for 10 minutes, permeabilized using 0.01% Triton X‐100 (Sigma Chemical Co.) in PBS for 30 seconds, and blocked for 30 minutes in PBS containing 0.2% normal rabbit serum and 1% BSA. The cells were then incubated in the presence of a monoclonal mouse antihuman IL‐18R‐alpha antibody (R&D, MAB 840) at a 1:200 dilution (diluted in PBS containing 1% BSA and 2% normal bovine serum) for 1 hour at 25°C. Then cells were incubated sequentially with goat anti‐rabbit immunoglobulins conjugated with FITC Fluor 568 (Invitrogen Corp.) for 60 minutes at 25°C. Cells on slides were visualized using a confocal laser‐scanning microscope (Leica Microsystems). A positive reaction was demonstrated by a green fluorescence. Negative controls were incubated with 2.5% BSA instead of the primary antibody.^30^

### 2.4 Cell viability and proliferation assay

Theca cells were seeded into 96‐well plates and cultured overnight with McCoy's medium containing 0.1% BSA. After attachment, cells were pretreated with IL‐18 (300 pg/mL) for 1 hour followed by LH (150 pg/mL) for 24 hours. After the treatment periods, cell viability was determined by MTT assay.^31^ For cell proliferation assay, TCs were treated with 10 μmol/L bromodeoxyuridine/5‐bromo‐2’‐deoxyuridine (BrdU) in the presence or absence of IL‐18 (0, 10, 30, 100, 300, 500 and 1000 pg/mL), cell proliferation was measured using an enzyme‐linked immunosorbent assay after 48 hours (Cell Proliferation ELISA, BrdU [colorimetric], Roche Applied Science), according to the manufacturer's recommendations. The absorbance was measured at 450 nm with a Thermo Lab Systems plate reader (Thermo Fischer Scientific) and Ascent Software for Multiskan equipment. Cell proliferation was normalized to the control condition of each culture. The results are expressed as mean ± standard error of the mean (SEM) of four independent experiments with three replicates per condition.

### 2.5 Steroid immunoassays

Steroid concentrations in cell culture media were determined by ELISA. The 17‐hydroxyprogesterone (17OHP4) assay had a detection limit of 20 pg/mL and intra‐ and inter‐assay CVs were 8% and 10% respectively. The androstenedione (A2) had a detection limit of 30 pg/mL and intra‐ and inter‐assay CVs were 7% and 10% respectively. The results, expressed in ng 17OHP4/pg A2 per μg protein, were then normalized to the control condition of each experiment and presented as mean ± SEM of four experiments with three replicates per condition.

### 2.6 RT‐PCR and quantitative (Q)‐PCR

Total RNA from TCs was extracted using the TRIzol reagent (Roche) according to the manufacturer's instructions. cDNA was synthesized from 1 μg of RNA using the AB High‐Capacity cDNA synthesis kit (Thermo Fisher Scientific) with random hexamers. Real‐time PCR was performed using SYBR green master mix on LightCycler^®^ 96 System (Roche Diagnostics Ltd). The sequences of the primers used were given in Table 1. qPCR was carried out using QuantiTect SYBR Green master mix (Qiagen) and an AB StepOne plus thermal cycler (Applied Biosystems). The PCR parameters used were a 10 seconds denaturation cycle at 95°C, followed by 40 cycles of 95°C for 10 seconds and 60°C for 30 seconds. PCR efficiencies were detected using a relative standard curve derived from diluted cDNA reaction mixture (a 2‐fold dilution series with five measuring points). The R2 values for all standard curves generated ranged between 0.997 and 0.999 and PCR efficiencies were between 90% and 110%. The β‐actin as an inner control was applied to normalize the mRNA expression results using $2^{‐\Delta \Delta C_{t}}$ method.

**TABLE 1:** Primer sequences

Gene	Sequences
CYP11A1	
Forward	5′‐GTGAAGATATTGACGAGTGT‐3′
Reverse	5′‐GATGAACCTGGCTGACTATCA‐3′
CYP17A1	
Forward	5′‐GCCAGGACCCAAGTGTGTTCTC‐3′
Reverse	5′‐AGACGGTGTTCGACTGAAGCCT‐3′
LH‐R	
Forward	5′‐AGGAAAATGCACGCCTGGAG‐3′
Reverse	5′‐GTGGCATCCAGGAGGTTGGT‐3′
β‐actin	
Forward	5′‐CTCTCAAGGGCATTCTAGGC‐3′
Reverse	5′‐TGAGAAAGTCGTTGAGG‐3′

Abbreviations: CYP11A1, cholesterol side‐chain cleavage enzyme; CYP17A1, P45017α‐hydroxylase; LH‐R, luteinizing hormone receptor.

### 2.7 Western blot

After supernatant removal and addition of lysis buffer to the cells (150 mmol/L NaCl, 1 mmol/L Tris, 1 mmol/L EDTA, 2 mmol/L ethylene glycol tetraacetic acid [EGTA], 2 mmol/L Na3VO4, 10 mmol/L NaF, 12 mmol/L NaH2PO4, 0.5% NP40 [v/v], 1% Triton X‐100 [v/v]), proteins were extracted on ice. Proteins (50 μg/lane) were separated by electrophoresis using 10% SDS‐PAGE and transferred to nitrocellulose membranes (Bio‐Rad) before immunoblot analysis. After transfer, the membranes were incubated with 5% nonfat milk powder at room temperature for 90 minutes and then with appropriate primary antibodies (Table 2) at 4°C overnight. Primary antibody binding was detected using horseradish peroxidase‐conjugated secondary antibody (1:5000 dilution). Detection was achieved with the ECL chemiluminescence kit (Millipore). The relative intensities of the bands were analysed by Quantiscan software (Biosoft). Values obtained for proteins were normalized with the GAPDH as a loading control.

**TABLE 2:** Antibodies used for Western blot analysis

Protein target	Primary antibody	Dilution used	Antibody type	Secondary antibody	Dilution used	Originated
P450scc	Mouse anti‐P450scc	1/1000	Polyclonal	anti‐mouse IgG	1/2000	Invitrogen, Carlsbad, CA, USA
P450c17	Mouse anti‐P450c17	1/1000	Polyclonal	anti‐mouse IgG	1/2000	Invitrogen, Carlsbad, CA, USA
LH‐R	Mouse anti‐LHR	1/1000	Monoclonal	anti‐mouse IgG	1/2000	Invitrogen, Carlsbad, CA, USA

Abbreviations: P450c17, P45017α‐hydroxylase; P450scc, cholesterol side‐chain cleavage enzyme.

### 2.8 Experimental design

#### 2.8.1 To evaluate the dose‐response effect of IL‐18 on proliferation of TCs

Theca cells were cultured for 48 hours in 10% FCS, washed twice with serum‐free mediums as described earlier, and 0, 10, 30, 100, 300, 500 and 1000 pg/mL of highly purified bovine IL‐18 were applied for different times (24, 48 and 72 hours) in the absence or presence of LH (150 pg/mL). The dose of LH was selected based on previous studies.^24^, ^32^ Media were changed and treatments replenished after one day. Cell viability was analysed by MTT assay. BrdU assay was utilized to assess the cell proliferation.

#### 2.8.2 To evaluate the dose‐response effect of IL‐18 on steroidogenesis of TCs

Theca cells were cultured for 48 hours in 10% FCS, washed twice with serum‐free medium, and 0, 10, 30, 100, 300, 500 and 1000 pg/mL of IL‐18 were applied for different times (24, 48, 72 hours) in the presence and absence of LH (150 pg/mL). LH was used at a final concentration of 150 pg/mL, shown previously to elicit maximal androstenedione secretion.^32^ Media were changed and treatments replenished after one day. Concentrations of A2 and 17OHP4 in conditioned media were determined by ELISA.

#### 2.8.3 To determine if IL‐18R abundance differed between small‐ and large‐follicle TCs

Based on surface diameter, theca cells were collected from small (3‐6 mm) and large (8‐22 mm) follicles as previously described.^26^, ^29^ This size classification was based on previous observations indicating that (a) follicles ≥8 mm in diameter have much greater androstenedione and estradiol concentrations than small follicles,^33^ (b) follicles that are destined to ovulate average 10 ± 2 mm surface diameter,^34^ and (c) similar classifications have been used previously to inventory follicles during bovine oestrous cycles.^35^ The IL‐18R protein was localized in TCs by IF. The fluorescence intensity was analysed by Image J to compare the difference of IL‐18R expression between large and small follicles.

#### 2.8.4 Effect of IL‐18 on thecal cell expression of steroidogenic pathway components

To evaluate the effects of IL‐18 on expression of key transcripts involved in steroidogenesis (CYP11A1, CYP17A1) and LH‐R, TC were cultured in 24‐well plates (250 000 cells/well) and exposed to fixed concentrations of IL‐18 (1000 ng/mL) in the presence and absence of an optimal concentration of LH (150 pg/mL). At the end of culture, media were removed and cell lysates were prepared for total RNA or protein extraction and RT‐qPCR or Western blot analysis.

#### 2.8.5 Can IL‐18BP neutralize the effect of IL‐18 on steroidogenesis and/or proliferation of TCs?

Since IL‐18BP has been shown to antagonize the effect of IL‐18, we evaluated whether IL‐18BP can neutralize the stimulation effects of IL‐18 on TCs proliferation and steroidogenesis. TCs were treated with different concentration of IL‐18BP (1, 10, 100, and 1000 ng/mL) for 4, 8, 16 and 24 hours in the presence of IL‐18 (1000 pg/mL). At the end of culture, BrdU assay was utilized to assess the cell proliferation and steroid concentrations in conditioned media were determined by ELISA.

### 2.9 Statistical analysis

All data were analysed using SPSS Statistics 22 software (IBM). A *P* value <0.05 was considered statistically significant. The effects of the various treatments on TCs proliferation, hormone secretion and gene expression were evaluated by one‐ and /or two‐way ANOVA due to a non‐normal distribution (Shapiro test) and non‐homogeneous variances (Levene test). Steroid concentrations were log‐transformed prior to statistical analysis to reduce the heterogeneity of variance. RT‐qPCR data were analysed as ΔΔCt values (ie, log2 values) before being converted to fold‐difference values for graphical presentation of relative transcript abundance. The data are represented as mean ± SEM, and all experiments were repeated at least three times independently, with similar results. Blots are representative of one experiment, and graphs represent the mean ± SEM of three replicates.

## 3 RESULTS

### 3.1 Purification of TCs

**FIGURE 1:**
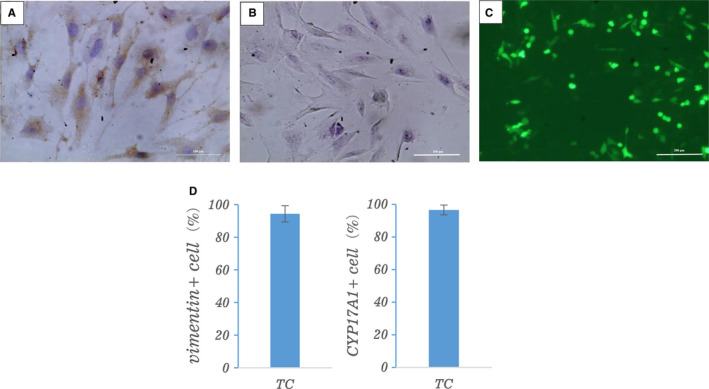
The purity of theca cells (TCs). TC cells were immuno‐stained brown with vimentin antibody as shown in image of (A) and were negative staining of cytokeratin antibody as shown in image of (B). Scale bars represent 100 μm. C, CYP17A1 immunofluorescent staining of theca cells after 1 or 2 d of culture in FCS. Scale bars represent 200 μm. D, Proportion of vimentin+ and CYP17A1+ in cultured thecal cells from immunohistochemistry and immunofluorescent staining, respectively

To evaluate the purity of isolated TCs and rule out fibroblast contamination, TCs were incubated with rabbit anti‐vimentin (1:100; Abcam) and rabbit anti‐cytokeratin (1:100; Abcam), and CYP17A1 was used for marker for TCs. Immunohistochemical staining showed that vimentin staining was positive in TCs (Figure 1A,D), indicating that vimentin was expressed in the cells, while cytokeratin staining was negative (Figure 1B). IF staining of follicle TCs revealed that over 95% of attached cells stained positive for CYP17A1 protein after 2‐day exposure to 10% FCS (Figure 1C,D).

### 3.2 Expression of IL‐18R in bovine TCs

**FIGURE 2:**
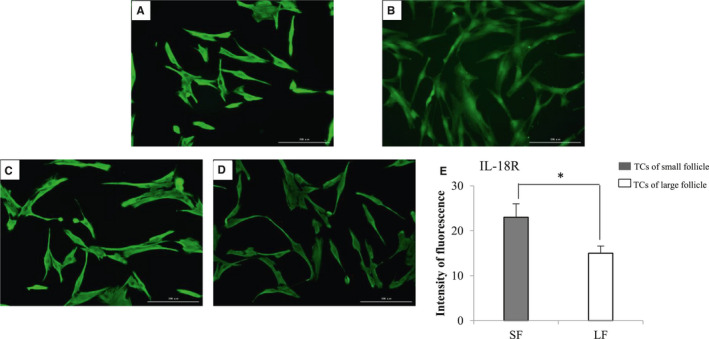
Immunofluorescence demonstration of IL‐18R. A, Localization of IL‐18R on the surface of TCs. B, TCs without the primary antibody as negative control. C, Expression of IL‐18R in TCs of small follicles. D, Expression of IL‐18R in TCs of large follicles. Scale bars represent 200 μm. E, The fluorescence intensity of IL‐18R analysed by ImageJ (the values represent a mean value at each time point based on multiple ovaries) shows that the intensity of green fluorescence in TC cells increased in small follicles than in large follicles. Student's *t* test was used, and the data are expressed as mean ± SEM (n = 4). *P < .05

To further reveal the manner of IL‐18 functions in follicles, we localized IL‐18R protein in TCs by IF. By means of Immunofluorescence, we were able to prove that bovine follicular TCs express IL‐18R. Figure 2A demonstrates the immunoreactive signal of IL‐18R was expressed in both the membrane and cytoplasm of theca cells, and the expression was mainly in the cell membrane. Bovine TCs from small and large follicles both expressed the IL‐18R. In small follicle (Figure 2C), the expression of IL‐18R was significantly higher than that in large follicle (Figure 2D). The fluorescence intensity was analysed by Image J and the results also showed that the expression of IL‐18R decreased gradually during follicular development (Figure 2E). The present data suggested that IL‐18 from TCs or serum could mediate the biological effects in the cells. Furthermore, IL‐18 may play an essential role in regulation of the growth and development of the follicles via an intrafollicular paracrine/autocrine manner.

### 3.3 Effect of IL‐18 on the proliferation of TCs

**FIGURE 3:**
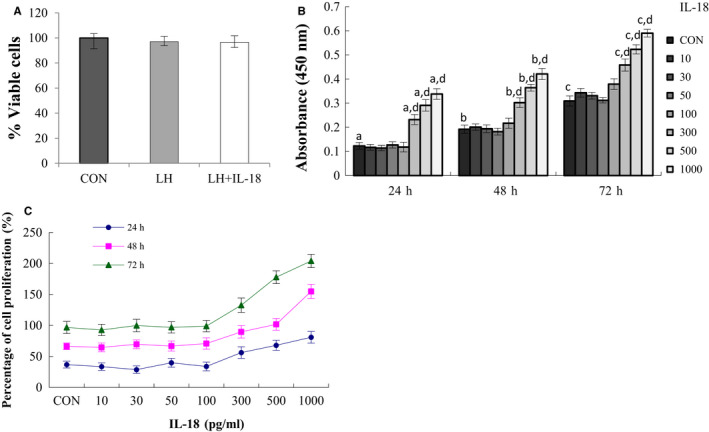
Effect of IL‐18 on LH‐induced cell proliferation. A, The viability of the cells was assessed by MTT assay. TCs were isolated by collagenase digestion and cells were plated with McCoy's medium. After 24‐h attachment, cells were pretreated without or with IL‐18 (300 pg/mL) for 1 h prior to treatment for 24 h with LH (150 pg/mL). Control groups received vehicle DMSO. Results are expressed as the percentage of viable cells compared with control. B, Dose‐ and time‐dependent effects of IL‐18 on the cell proliferation was assessed by BrdU incorporation. Cells were incubated with or without increasing concentrations of IL‐18 for 24, 48 and 72 h. Data represent the mean ± SEM of at least four independent experiments. a‐c, *P *< .05 vs control, d, Significant differences (*P* < .05) compared between groups. C, Cell proliferation percentage. Cell proliferation rate = [(absorbance value of experimental group/absorbance value of control group) − 1] × 100%

The initial experiments examined toxicity of IL‐18 treatment in cultured TCs using cell viability assay. To test this, cultured TCs were preincubated with or without IL‐18 (300 pg/mL) for 1 hour followed by treatment with LH for 24 hours. Cell viability was analysed by MTT assay. The results presented in Figure 3A show that IL‐18 treatment at 300 pg/mL concentration did not reduce cell viability compared with control or LH treatment group.

To identify the exact effect of IL‐18 on the TC proliferation, BrdU assay was utilized to assess the cell proliferation. Cultured TCs isolated from bovine ovaries were treated with different concentration of IL‐18 (0, 10, 30, 100, 300, 500 and 1000 pg/mL) for 24, 48, and 72 hours in the presence of BrdU. As shown in Figure 3B, the results showed that treatment with IL‐18 in TCs caused a biphasic dose‐response, with 10‐100 pg/mL were without effect, whereas 300‐1000 pg/mL in dose and time‐dependent promotes the incorporation of BrdU into newly synthesized DNA in TCs (*P* < .05), suggesting that IL‐18 stimulates TCs proliferation. The curve of cell proliferation percentage showed that at the highest dose tested, IL‐18 increased cell numbers by 1.5‐ to 2‐fold (Figure 3C).

### 3.4 Activation of JNK by IL‐18‐induced cell proliferation in bovine theca cells

**FIGURE 4:**
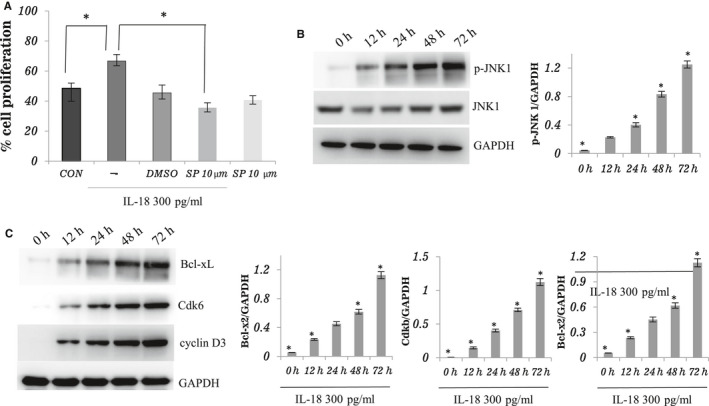
Involvement of JNK pathway for IL‐18‐induced cell proliferation in bovine theca cells. A, Theca cells were pretreated with or without SP600125 (10 μmol/L) for 1 h before being treated with IL‐18, and then incubated with 300 pg/mL IL‐18 for 24 h in serum‐free media. DMSO was used as the control for SP600125. After incubation, cells were harvested and the percentage of cell proliferation was detected by BrdU incorporation. Three independent experiments were carried out in triplicate; bars, mean ± SEM. *P < .05. B and C, Theca cells were treated with IL‐18 (300 pg/mL) for 0, 12, 24, 48 and 72 h. The cells were lysed, and the level of Bcl‐xL, Cdk6, cyclin D3 and JNK 1 phosphorylation was examined by Western blot. The total JNK 1 was detected to confirm the equal volume of the cell lysates. Three independent experiments were carried out in triplicate; bars, mean ± SEM. *P < .05 compared between groups

The JNK signal pathway appears to play a significant role in IL‐18 signalling. Therefore, this study examined whether or not IL‐18 actually activates this kinase in bovine theca cells. The theca cells were treated with high‐dose IL‐18 during a 3‐d culture results in strikingly enhanced activation of JNK, which correlated with increased expression of the anti‐apoptotic factor Bcl‐xL, and cell cycle–related factors Cdk6 and cyclin D3 (Figure 4B,C). Furthermore, pretreatment of IL‐18–stimulated cultures with the JNK inhibitor SP600125 revealed that IL‐18–dependent increases in cell proliferation is particularly sensitive to JNK inhibition (Figure 4A), suggesting that enhanced JNK activation may be the key molecular mechanism of IL‐18 stimulation on proliferation of theca cell.

Direct evidence for JNK activation was provided by immunoblotting the whole cell lysated with anti‐phospho‐JNK antibody after the IL‐18 treatment. As shown in Figure 4B, the IL‐18 treatment increased the level of JNK phosphorylation. This suggests that IL‐18 enhances theca cell proliferation by activating JNK.

### 3.5 Effect of IL‐18 on basal and LH‐induced steroid secretion by TCs

**FIGURE 5:**
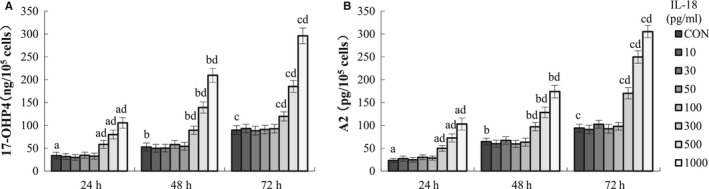
Effect of IL‐18 on TC steroid secretion. Cells were incubated with or without increasing concentrations of IL‐18 for 24, 48 and 72 h. The A2 (A) and 17OHP4 (B) concentrations were measured in culture media, and the value was normalized by the protein concentration in each well. Data are expressed as ng 17OHP4/pg A2 per μg protein and normalized to the control condition. Data represent the mean ± SEM of at least four independent experiments. a‐c, *P* < .05 vs control, d, significant differences (*P* < .05) compared between groups

To examine the effects of IL‐18 on steroid biosynthesis, cultured TCs isolated from bovine ovaries were treated with different concentrations of IL‐18 (0, 10, 30, 100, 300, 500 and 1000 pg/mL) for 24, 48, and 72 hours. The effects of IL‐18 treatment on basal and LH‐stimulated androstenedione (A2) and 17‐hydroxyprogesterone (17OHP4) production are presented in Figure 5. The results showed that treatment with IL‐18 in TCs caused a biphasic dose‐response, with 10‐100 pg/mL were without effect, whereas 300‐1000 pg/mL in dose and time‐dependent promotes the secretion of A2 and 17OHP4 (*P* < .05).The Figure 5 showed that at the highest dose of IL‐18 tested (1000 pg/mL) for 72 hours, the stimulated effect of IL‐18 on the secretion of A2 and 17OHP4 of TCs reached the peak (*P* < .05).

### 3.6 Effects of IL‐18 on TC expression of steroidogenesis‐related transcripts

To test the cellular mechanisms underlying IL‐18 stimulation of A2 and 17OHP4 biosynthesis, the ability of IL‐18 to alter abundance of LHR, CYP11A1, and CYP17A1 mRNA and protein were analysed in cultured TCs.

**FIGURE 6:**
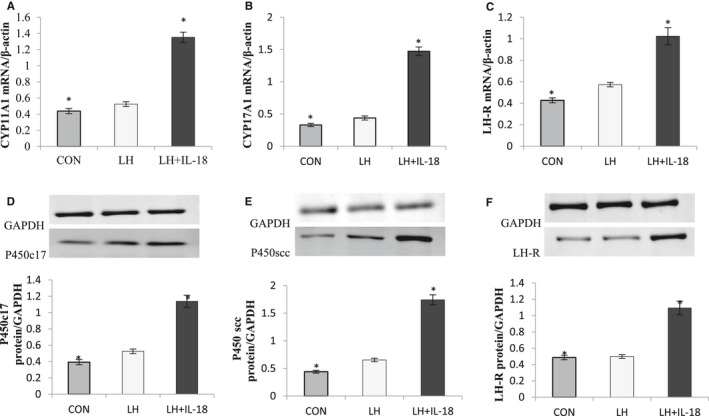
Effect of IL‐18 on steroidogenic enzyme expression. A‐C, The expression of three genes involved in steroidogenesis (CYP17A1, CYP11A1 and LH‐R) was determined in TC after 72‐h culture in complemented serum‐free McCoy's 5A media in presence or absence of IL‐18 (1000 pg/mL). Total mRNA was extracted from TC and reverse transcribed, and qPCR was performed. The housekeeping gene β‐actin was used to normalize gene expression. D‐F, The expression of the steroidogenic enzyme proteins (P450c17, P450scc and LH‐R) was determined in TC after 72‐h culture in the presence or absence of IL‐18 (1000 pg/mL). Proteins were then extracted and separated using electrophoresis on 4%‐12% (w/v) sodium dodecyl sulphate polyacrylamide gels. After electrotransfer to nitrocellulose membranes, the proteins were probed with P450c17, P450scc and LH‐R and GAPDH antibodies. The results are expressed as mean ± standard error of the mean (SEM) of six independent cultures. *Indicates a tendency (*P* < .05)

Figure 6 shows the effects of IL‐18 on the relative expression of steroidogenesis‐related enzyme by TCs cultured under basal and LH‐stimulated conditions. One dose level of the treatment (1000 pg/mL IL‐18) was selected based on optimal responses in the dose‐response experiment (Figure 5). Our findings showed that the expression levels of CYP11A1, CYP17A1 and LH‐R mRNA, which are crucial for androgen synthesis, were significantly increased (*P* < .05), after the cells were treated with IL‐18 (Figure 6A‐C). Their expression tendency at protein levels was similar to that of mRNA (Figure 6D‐F). The current results have given evidence that IL‐18 may play a promoting role in TC proliferation and steroidogenesis in vitro during the follicular growth and development.

### 3.7 Effects of IL‐18BP on IL‐18‐induced proliferation and steroidogenesis of TCs

To examine whether IL‐18BP can neutralize the effect of IL‐18 on steroidogenesis and/or proliferation of TCs, cultured TCs isolated from bovine ovaries were treated with different concentration of IL‐18BP (1, 10, 100, and 1000 ng/mL) for 4, 8, 16 and 24 hours in the presence of IL‐18 (1000 pg/mL). One dose level of the treatment (1000 pg/mL IL‐18) was selected based on optimal responses in the dose‐response experiment (Figure 5).

**FIGURE 7:**
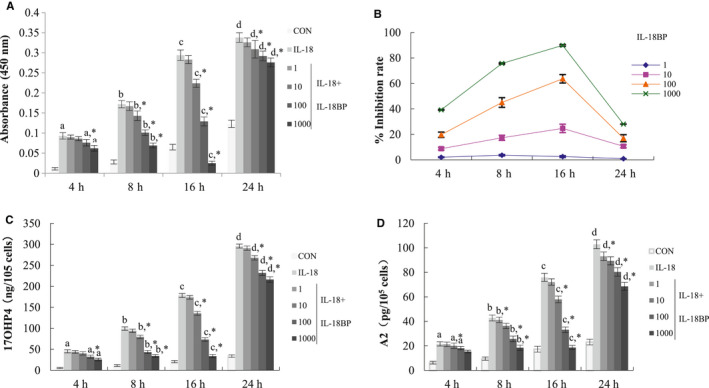
Effects of IL‐18BP on IL‐18‐induced proliferation and steroid hormone secretion of TCs. A, Dose‐ and time‐dependent effects of IL‐18BP inhibited IL‐18‐induced cell proliferation was assessed by BrdU incorporation. TCs were pretreated with different concentrations of IL‐18BP (1, 10, 100 and 1000 ng/mL) for 30 min, and then, 1000 ng/mL IL‐18 was added for different time. B, Inhibition rates of different concentrations of IL‐18BP on Il‐18‐induced proliferation of thecal cells. Inhibition rate = [(absorbance value of experimental group/absorbance value of IL‐18 group) − 1] × 100%. C,D, Dose‐ and time‐dependent effects of IL‐18BP inhibited Il‐18‐induced steroid secretion in TCs. The A2 (C) and 17OHP4 (D) concentrations were measured in culture media, and the value was normalized by the protein concentration in each well. Data represent the mean ± SEM of at least four independent experiments. a‐d, *P* < .05 vs IL‐18 group. *Significant differences (*P* < .05) compared between groups

As shown in Figure 6A, the results showed that treatment with IL‐18BP caused a biphasic dose‐response, with 1 ng/mL was without effect, whereas 10‐1000 ng/mL had significant inhibitory effect on the proliferation of TCs induced by IL‐18 in dose and time‐dependent (*P* < .05). The inhibitory effect reached the peak at 16 hours (*P* < .05, Figure 7A), and the inhibitory effect was obviously weakened at 24 hours. The curve of cell proliferation inhibition rate showed that at the highest dose of IL‐18BP tested (1000 ng/mL) for 16 hours, the effect of IL‐18 on the proliferation of TCs was reduced to about 10% (Figure 7B). The effects of IL‐18BP treatment on IL‐18 induced A2 (Figure 7C) and 17OHP4 (Figure 7D) production were similar to that of cell proliferation. The results suggest that IL‐18BP can reverse the effect of IL‐18 on proliferation and steroidogenesis of TCs.

## 4 DISCUSSION

The TCs are one of the most important cell types in the follicles and have a key role during follicular growth and atresia. The TCs consist of three to five layers of differentiated cells that surround the basal lamina and secrete steroid.^19^ The function of TCs includes synthesizing androgens, promoting the function of GCs, oocyte development, and providing structural support for the follicle.^36^ In the present study, we evaluated the effects of IL‐18 on TC functions, include TC cell proliferation and steroidogenesis, aimed to examine the potential involvement of IL‐18 in regulating ovarian follicle function by utilizing a primary bovine theca interna cell culture model.

Several studies have revealed the impact of IL‐18 on cell proliferation. The research showed that IL‐18 not only induced IFN‐γ secretion, but also stimulated splenocyte proliferation with a induced dose‐dependent effect. Phenotypic analyses revealed that IL‐18 mainly affected the proliferation of CD4^+^ cells and identified two distinct mechanisms of IL‐18‐induced IFN‐γ secretion.^37^ In addition, with the synergistic effect of IL‐12 and IL‐15, IL‐18 can induce proliferation of human CIML NK cells, and this impact may through the Jak1/2 pathway.^38^ Victor et al identified IL‐18 as a cytokine that cooperates with IL‐15, to induce proliferation of human ILC3s, as well as induce and maintain IL‐22 production, and the mechanism of action is NF‐kB pathway activated by IL‐18 signalling.^39^ An increase in theca cell number and size is seen in pathological conditions, such as PCOS, which is the most common cause of infertility in women. The present studies provide new insights into the intracellular signalling cascade by which IL‐18 induces TCs proliferation. Our study revealed that high doses of IL‐18 stimulated the proliferation of TCs, indicating that the increase in theca cell seen under pathological conditions such as PCOS may be due to hyper activation of IL‐18. Therefore, based on these findings, we suggest that IL‐18 has a role in the thickness of the cortex, the increased ovary volume, and even the hyperplasia of follicle numbers, the tunica albuginea, and ovarian medulla observed in PCOS ovaries.

The present study revealed that bovine TCs expressed IL‐18R mRNA, suggesting that bovine theca cells are capable of responding to IL‐18. Moreover, IL‐18 stimulated the production of 17OHP4 and A2 in LH‐stimulated TCs, increasing the transcription of steroidogenic enzymes CYP11A1 and CYP17A1. These findings indicate that IL‐18 can locate on bovine theca cells and stimulate the steroidogenic function of theca cells.

We recently reported that the level of FF IL‐18 in PCOS patients was higher than that in the control women, which was consistent with the results in the blood.^40^ The results suggested that the inflammatory reaction occurred not only in the serum but also in the FF of the patients with PCOS. Furthermore, the IL‐18 levels were significantly higher in the FF than in serum, and the elevated FF IL‐18 levels have no correlation with the serum IL‐18 levels. This finding suggested a local production of IL‐18 in the microenvironment of PCOS ovary. Based on this report and the results of the present study, it can be assumed that IL‐18 might cause ovarian dysfunction and subsequent impaired fertility.

For the first time, the present experiments showed that presumed physiological concentrations of IL‐18 (ie, 10 to 100 pg/mL) were inactive in theca cell cultures. Although not known for bovine, the concentrations of IL‐18 in follicular fluid of mature, immature and atresia follicles were 26‐55.5, 23.4‐53.8 and 18.2‐56.7 pg/mL,^41^, ^42^ respectively, and the levels in the serum of normal women were 64 ± 17 pg/mL.^42^, ^43^ Beyond this concentration range, we observed that IL‐18 stimulated theca cell steroidogenesis and cell proliferation in a dose‐dependent manner, suggesting a possible regulatory role in follicular development.

The increase of steroid production in PCOS theca cells is associated with increased gene expression of several steroidogenic enzymes important for androgen biosynthesis, including CYP11A1, CYP17A1, HSD3B2 and StAR.^14^ CYP11A1 gene encodes the P450scc enzyme that is the first and rate‐limiting enzyme in the steroidogenic pathway, converting cholesterol to pregnenolone.^44^ Pregnenolone is the precursor of progesterone, and thus a higher pregnenolone concentration may lead to increased progesterone production and secretion. Assessing the expression and activity of enzymes could highlight whether IL‐18 affects steroidogenesis through these enzymes. Based on our mRNA studies, IL‐18 treatment increased the mRNA expression of CYP11A1 and CYP17A1 and the corresponding protein expression, which suggests that IL‐18 appears to increase progestin precursor formation and subsequently increases the androgen formation.

Luteinizing hormone also is needed to support follicular development, since this gonadotropin stimulates progestogen and androgen production by the LH‐responsive steroidogenic TCs of the multilayered growing follicles.^45^ Steroidogenesis and the expression of steroidogenesis‐related genes in TCs are primarily under control of the LH/LHR pathway. Consistent with this fact, serum concentrations of each steroid hormone and the expression of almost steroidogenesis‐related genes decreased in response to a reduction of LH and LHR expression. In the present study, IL‐18 up‐regulated the mRNA expression of LHR in theca cells, which may support the possibility of a distinct mechanism of IL‐18 effects of theca cell function. It might cause improper follicular maturation and impaired ovarian activity.

With regard to the cytokine receptors examined, the present findings that IL‐18R abundance was greater in small‐follicle rather than large‐follicle TCs, and the IL‐18R expression in TC declined to a low level in large follicles, suggesting attenuation of IL‐18 signalling at this stage and immature undifferentiated follicles may be more sensitive to IL‐18 than those of mature differentiated follicles. IL‐18 may preferentially stimulate theca cell during early follicle development. The significant changes in the IL‐18R levels with follicle size, probably reflects the important changing roles of IL‐18 in follicular growth at the stages examined. This result indicated that IL‐18 exerts an indispensable role in the growth and development of the ovarian follicles. This is also consistent with the ovarian pathological morphology of PCOS, with the main manifestations of excessive proliferation of thecal cells, overdevelopment of small follicles and anovulation.

IL‐18 binding protein (IL‐18BP) is a secreted 40 kDa glycoprotein which possesses a high affinity to IL‐18. This constitutively expressed soluble protein prevents the binding of mature IL‐18 to its receptor and inhibits IL‐18 biological activity. Our data demonstrated that IL‐18 plays a stimulatory role in TC proliferation and steroidogenesis as well as in the expression of steroidogenic enzyme CYP17A1 and CYP11A1 mRNA and proteins within the TCs in vitro, and IL‐18BP can neutralize these effects of IL‐18. It is expected to open up a new way for the treatment of PCOS.

The data reported in this manuscript were obtained from animal cell models and are not applicable to daily medical practice. Further in‐depth studies in other species, including whole animal models, are required to confirm and extend these in vitro observations based on bovine ovarian cell culture models. There is a need for additional studies to verify the effects observed in this study, and further in vitro studies are needed to investigate the mechanisms through which IL‐18 exerts its effects.

In summary, for the first time, we reported that IL‐18 increased basal and/or LH‐induced cell proliferation and both androstenedione and 17‐hydroxyprogesterone secretion, while IL‐18BP reversed these effects. IL‐18 also up‐regulated the expression of key steroidogenic‐related genes in androstenedione secretion, including CYP17A1 and CYP11A1. The present study provides new evidence for IL‐18‐dependent regulation of proliferation and steroidogenesis in TCs that may influence follicle development. It is now convincingly suggested that IL‐18 may serve as a critical regulator of cell proliferation, differentiation and steroidogenesis in the bovine TCs of ovarian prehierarchical follicles. But additional research is needed to understand the mechanism of action of IL‐18 in theca cells, as well as its precise role in folliculogenesis. Further studies are needed to demonstrate a connection between inflammatory factors and PCOS pathogenesis. By verifying the involvement of inflammatory factors in the pathogenesis of PCOS, new methods may be developed for PCOS treatment, such as blocking therapeutic targets including inflammatory factors and signalling pathways.

## CONFLICTS OF INTEREST

The authors have no conflicts of interest to disclose.

## AUTHOR CONTRIBUTIONS

**Hong yuan Zhang:** Data curation (lead); Writing‐original draft (lead); Writing‐review & editing (lead). **Fu fan Zhu:** Data curation (equal); Investigation (equal); Writing‐original draft (equal). **Ying jun Zhu:** Formal analysis (lead); Investigation (lead); Project administration (lead). **Xu Chen:** Conceptualization (equal); Resources (equal); Software (equal). **Yuan jing Hu:** Methodology (equal); Validation (equal); Writing‐review & editing (equal).

## Data Availability

The data sets are available under reasonable request.
